# Supplementary material for: Performance evaluation of national healthcare systems in the prevention and treatment of non-communicable diseases in sub-Saharan Africa
Source: PLoS One. 2023 Nov 16;18(11):e0294653. doi: 10.1371/journal.pone.0294653 (PMC10653434; doi:10.1371/journal.pone.0294653)
Supplement: S1 Appendix — (DOCX) [file pone.0294653.s001.docx]

# SUPPLEMENTARY MATERIALS

## S1 Appendix: Descriptive statistics on all the variables

| Variable | Mean | Std. Dev | Min | Max |
| --- | --- | --- | --- | --- |
| *Inputs* |  |  |  |  |
| NCDs spending per capita (PPP) | 98.09 | 196.37 | 0.76 | 1017.96 |
| Health workers per 10,000 people | 5.18 | 3.23 | 1.35 | 17.44 |
| *Outputs* |  |  |  |  |
| NCDs mortality rate per 100,000 people | 634.46 | 100.70 | 429.80 | 968.50 |
| NCDs DALYs per 100,000 people | 23146.83 | 2196.62 | 18566.20 | 30632.50 |
| UHC on NCDs | 63.95 | 8.12 | 44.00 | 80.00 |
| *Environmental variables* |  |  |  |  |
| Smoking per capita | 0.12 | 0.05 | 0.04 | 0.24 |
| Alcohol use per capita (in liters) | 4.73 | 3.63 | 0.00 | 14.51 |
| Pollution from solid fuel (per m^3^ of air) | 0.74 | 0.29 | 0.01 | 0.99 |
| Proportion of urban population | 44.72 | 17.29 | 15.97 | 89.74 |
| Income (GDP per capita, PPP) | 5332.98 | 5726.89 | 753.49 | 28297.50 |
| Governance quality | -0.52 | 0.59 | -2.12 | 0.80 |
| NCDs external funding (% of TEHF**^1^**) | 3.71 | 7.05 | 0.00 | 56.65 |
| NCDs private domestic funding per capita | 20.65 | 45.51 | 0.04 | 266.76 |

**^1^**TEHF = Total External Health Funding
