# Supplementary material for: Performance evaluation of national healthcare systems in the prevention and treatment of non-communicable diseases in sub-Saharan Africa
Source: PLoS One. 2023 Nov 16;18(11):e0294653. doi: 10.1371/journal.pone.0294653 (PMC10653434; doi:10.1371/journal.pone.0294653)
Supplement: S2 Appendix — (DOCX) [file pone.0294653.s002.docx]

# SUPPLEMENTARY MATERIALS

## S2 Appendix: Bias-corrected technical efficiency scores of SSA countries (2015 – 2019)

| Country | 2015 | 2016 | 2017 | 2018 | 2019 | Average | Rank |
| --- | --- | --- | --- | --- | --- | --- | --- |
| Benin | 0.928 | 0.916 | 0.952 | 0.950 | 0.978 | 0.945 | 7 |
| Botswana | 0.692 | 0.692 | 0.700 | 0.708 | 0.716 | 0.702 | 34 |
| Burkina Faso | 0.947 | 0.962 | 0.969 | 0.970 | 0.969 | 0.963 | 3 |
| Cabo Verde | 0.967 | 0.973 | 0.965 | 0.903 | 0.906 | 0.943 | 8 |
| Central African Rep. | 0.772 | 0.731 | 0.715 | 0.693 | 0.712 | 0.725 | 33 |
| Comoros | 0.909 | 0.912 | 0.908 | 0.928 | 0.933 | 0.918 | 12 |
| Congo Republic | 0.764 | 0.786 | 0.805 | 0.832 | 0.830 | 0.804 | 28 |
| Côte d'Ivoire | 0.824 | 0.842 | 0.855 | 0.860 | 0.874 | 0.851 | 21 |
| DR. Congo | 0.891 | 0.920 | 0.922 | 0.926 | 0.932 | 0.918 | 10 |
| Eswatini | 0.802 | 0.782 | 0.792 | 0.789 | 0.795 | 0.792 | 30 |
| Ethiopia | 0.976 | 0.976 | 0.973 | 0.972 | 0.971 | 0.973 | 2 |
| Gabon | 0.777 | 0.774 | 0.779 | 0.780 | 0.781 | 0.778 | 32 |
| Ghana | 0.852 | 0.865 | 0.902 | 0.915 | 0.928 | 0.892 | 17 |
| Guinea | 0.824 | 0.825 | 0.851 | 0.857 | 0.853 | 0.842 | 23 |
| Kenya | 0.907 | 0.914 | 0.916 | 0.918 | 0.918 | 0.915 | 14 |
| Liberia | 0.950 | 0.951 | 0.962 | 0.973 | 0.927 | 0.953 | 6 |
| Malawi | 0.891 | 0.907 | 0.930 | 0.951 | 0.968 | 0.929 | 9 |
| Mali | 0.841 | 0.858 | 0.956 | 0.886 | 0.887 | 0.886 | 18 |
| Mauritania | 0.903 | 0.903 | 0.907 | 0.915 | 0.922 | 0.910 | 15 |
| Mauritius | 0.801 | 0.806 | 0.805 | 0.805 | 0.806 | 0.805 | 27 |
| Mozambique | 0.895 | 0.930 | 0.895 | 0.935 | 0.920 | 0.915 | 13 |
| Namibia | 0.782 | 0.785 | 0.788 | 0.790 | 0.795 | 0.788 | 31 |
| Niger | 0.964 | 0.968 | 0.978 | 0.982 | 0.982 | 0.975 | 1 |
| Nigeria | 0.885 | 0.890 | 0.902 | 0.908 | 0.917 | 0.900 | 16 |
| Sao Tome and Principe | 0.961 | 0.972 | 0.967 | 0.961 | 0.955 | 0.963 | 4 |
| Senegal | 0.913 | 0.913 | 0.925 | 0.924 | 0.914 | 0.918 | 11 |
| Seychelles | 0.831 | 0.826 | 0.827 | 0.826 | 0.824 | 0.827 | 25 |
| Sierra Leone | 0.791 | 0.797 | 0.796 | 0.794 | 0.803 | 0.796 | 29 |
| South Africa | 0.794 | 0.809 | 0.822 | 0.854 | 0.871 | 0.830 | 24 |
| Togo | 0.845 | 0.845 | 0.863 | 0.876 | 0.875 | 0.861 | 20 |
| Uganda | 0.857 | 0.857 | 0.881 | 0.896 | 0.910 | 0.880 | 19 |
| Tanzania | 0.954 | 0.956 | 0.963 | 0.971 | 0.969 | 0.962 | 5 |
| Zambia | 0.797 | 0.823 | 0.860 | 0.874 | 0.888 | 0.848 | 22 |
| Zimbabwe | 0.820 | 0.820 | 0.831 | 0.826 | 0.834 | 0.826 | 26 |
| **Average** | **0.862** | **0.867** | **0.878** | **0.881** | **0.884** | **0.873** |  |
