# Supplementary material for: Performance evaluation of national healthcare systems in the prevention and treatment of non-communicable diseases in sub-Saharan Africa
Source: PLoS One. 2023 Nov 16;18(11):e0294653. doi: 10.1371/journal.pone.0294653 (PMC10653434; doi:10.1371/journal.pone.0294653)
Supplement: S3 Appendix — (DOCX) [file pone.0294653.s003.docx]

# SUPPLEMENTARY MATERIALS

## S3 Appendix: Variance inflation factor test for multicollinearity

| Variable | VIF | 1/VIF |
| --- | --- | --- |
| Pollution from solid fuel | 8.21 | 0.121802 |
| GDP per capita | 6.07 | 0.164798 |
| Private domestic funding for NCDs per capita | 3.38 | 0.295995 |
| Governance quality | 2.17 | 0.460982 |
| Urbanization (% of urban population) | 2.09 | 0.478004 |
| Smoking per capita | 1.77 | 0.565007 |
| External funding for NCDs (% of total external health funding) | 1.27 | 0.786702 |
| Alcohol use per capita | 1.16 | 0.864122 |
| **Mean VIF** | **3.26** |  |
