# Supplementary material for: Performance evaluation of national healthcare systems in the prevention and treatment of non-communicable diseases in sub-Saharan Africa
Source: PLoS One. 2023 Nov 16;18(11):e0294653. doi: 10.1371/journal.pone.0294653 (PMC10653434; doi:10.1371/journal.pone.0294653)
Supplement: S4 Appendix — (DOCX) [file pone.0294653.s004.docx]

# SUPPLEMENTARY MATERIALS

**S4 Appendix:** Pair-wise Pearson’s correlation coefficients

|  | Smoking | Alcohol | Pollution | Urbanization | GDP pc | Gov_qual | External | Private |
| --- | --- | --- | --- | --- | --- | --- | --- | --- |
| Smoking | 1 |  |  |  |  |  |  |  |
| Alcohol | 0.0907 | 1 |  |  |  |  |  |  |
| Pollution | -0.5105 | -0.328 | 1 |  |  |  |  |  |
| Urbanization | 0.1615 | 0.2074 | -0.6317 | 1 |  |  |  |  |
| GDP pc | 0.6191 | 0.3247 | -0.8863 | 0.4677 | 1 |  |  |  |
| Governance | 0.3761 | 0.2512 | -0.6462 | 0.3157 | 0.6752 | 1 |  |  |
| External | 0.2649 | 0.0717 | -0.3995 | 0.1400 | 0.4548 | 0.2235 | 1 |  |
| Private | 0.6478 | 0.192 | -0.7261 | 0.2285 | 0.7812 | 0.6129 | 0.3820 | 1 |
