# Supplementary material for: Performance evaluation of national healthcare systems in the prevention and treatment of non-communicable diseases in sub-Saharan Africa
Source: PLoS One. 2023 Nov 16;18(11):e0294653. doi: 10.1371/journal.pone.0294653 (PMC10653434; doi:10.1371/journal.pone.0294653)
Supplement: S5 Appendix — (DOCX) [file pone.0294653.s005.docx]

# SUPPLEMENTARY MATERIALS

## S5 Appendix: Tobit regression results ^1^

| Variables | Estimated  Coefficient | Standard Error | 95% confidence interval | |
| --- | --- | --- | --- | --- |
|  |  |  | Lower | Upper |
| Constant | 1.461*** | 0.118 | 1.228 | 1.695 |
| Smoking per capita | -0.618*** | 0.100 | -0.816 | -0.421 |
| Alcohol use per capita | -0.001 | 0.001 | -0.003 | 0.002 |
| Pollution from solid fuel | -0.078** | 0.039 | -0.154 | -0.001 |
| Governance quality | 0.050*** | 0.011 | 0.028 | 0.071 |
| Urbanization | -0.001*** | 0.000 | -0.002 | 0.000 |
| Log of GDP per capita | -0.046*** | 0.011 | -0.069 | -0.024 |
| External funding for NCDs | 0.002** | 0.001 | 0.000 | 0.004 |
| Log of Private domestic funding for NCDs | 0.000 | 0.000 | -0.001 | 0.000 |
| Sigma ($\hat{\sigma_{\varepsilon}}$) | 0.003*** | 0.000 | 0.002 | 0.004 |

**^1^**Dependent variable: Bias-corrected efficiency scores$(i.e. 0<\hat{\theta}^{**}<1)$.

***and **represent statistical significance at levels 1 and 5%, respectively.
