# Supplementary material for: Performance evaluation of national healthcare systems in the prevention and treatment of non-communicable diseases in sub-Saharan Africa
Source: PLoS One. 2023 Nov 16;18(11):e0294653. doi: 10.1371/journal.pone.0294653 (PMC10653434; doi:10.1371/journal.pone.0294653)
Supplement: S6 Appendix — (DOCX) [file pone.0294653.s006.docx]

# SUPPLEMENTARY MATERIALS

## S6 Appendix: Descriptive statistics based on income-groups (2015 – 2019 averages)

|  | Input Variables | |  | Output Variables | | |
| --- | --- | --- | --- | --- | --- | --- |
| Income Groups | NCDs Spending **^a^** | Health Workers Density **^a^** |  | NCDs Mortality Rate **^a^** | NCDs DALYs | UHC on NCDs **^a^** |
| Full Sample | 113.7 | 5.346 |  | 638.4 | 23182 | 63.10 |
|  | (216.4) | (3.483) |  | (108.4) | (2320) | (8.318) |
| Low-Income | 12.61 | 3.532 |  | 652.5 | 23560 | 66.58 |
|  | (8.985) | (1.354) |  | (109.3) | (2466) | (6.862) |
| Lower-Middle | 41.52 | 4.919 |  | 622.9 | 22852 | 64.67 |
|  | (44.77) | (2.635) |  | (119.5) | (2441) | (6.376) |
| Upper-Middle | 384.5 | 7.871 |  | 682.2 | 23606 | 53.35 |
|  | (200.0) | (1.741) |  | (54.45) | (1643) | (3.573) |
| High-Income | 770.6 | 15.29 |  | 575.7 | 22345 | 48.20 |
|  | (136.0) | (1.386) |  | (5.868) | (123.4) | (6.088) |

Values in parentheses are standard deviations

**^a^** Kruskal-Wallis test indicates a statistically significant difference at 1% across the income groups.
