# Supplementary material for: Performance evaluation of national healthcare systems in the prevention and treatment of non-communicable diseases in sub-Saharan Africa
Source: PLoS One. 2023 Nov 16;18(11):e0294653. doi: 10.1371/journal.pone.0294653 (PMC10653434; doi:10.1371/journal.pone.0294653)
Supplement: Attachment — Submitted filename: Response to Reviewers.docx [file pone.0294653.s008.docx]

Dear Editor,

Thank you for allowing us to resubmit a second revised draft of Manuscript Number PONE-D-23-12526R1 titled “Performance Evaluation of National Healthcare Systems in the Prevention and Treatment of Non-Communicable Diseases: Data Envelopment Analysis” to *PLOS ONE* journal. We appreciate the time and effort that you and the two reviewers dedicated to providing valuable feedback on our manuscript. We are grateful to you and the reviewers for the insightful comments on our paper. We have been able to make changes to reflect most of the suggestions provided by you and the reviewers. Here is a point-by-point response to all comments and concerns raised.

**Editor’s Comments and Our Responses:**

**Comment 1**. Please ensure that your manuscript meets PLOS ONE's style requirements, including those for file naming.

**Response:** We have made the necessary changes to the manuscript to ensure that our paper conforms to PLOS ONE’s style and policy. All the files have also been named accordingly. We have deposited the updated minimal datasets underlying the results described in our manuscript as well as all the STATA 17 and DEAP 2.1 do files and other Excel files used in the new revised manuscript at Zenodo repository with URLs of https://zenodo.org/record/8151509 and DOI of <https://doi.org/10.5281/zenodo.8151509> which was used earlier. The data set and the do files would make it easy for anyone to replicate the results of the study. Besides, we have added an Excel file of the data set and the STATA do files as Supporting Information files.

**Reviewer #2 Comments and Our Responses:**

**Comment 1**:  If the authors have adequately addressed your comments raised in a previous round of review and you feel that this manuscript is now acceptable for publication, you may indicate that here to bypass the “Comments to the Author” section, enter your conflict-of-interest statement in the “Confidential to Editor” section, and submit your "Accept" recommendation. **Reviewer #2:** All comments have been addressed

**Response:** We thank Reviewer #2 for his time and effort to review our manuscript.

**Reviewer #3 Comments and Our Responses:**

**Comment 1:** Thank you very much for all your replies to my former comments. Nevertheless, there is still a general lack of statistical knowledge. Although pros and cons of the proposed approach are discussed properly, no solutions to the discussed drawbacks are proposed, applied, investigated. Mainly, statistical inference is a crucial aspect of any empirical analyses. A comparison with other approaches, SFA for example, is required.

**Response:** We have conducted a stochastic frontier analysis (SFA) using the same dataset and compared the results with the data envelopment analysis (DEA) approach that was initially used (see pages 7, 8, 19, 21, and 22).

**Comment 2:** Longitudinal and spatial dimensions do not refer to noise. Both must be included, as a main assumption of the modelling (i.e., independence across observations) is not tenable. You can easily include both even in the DEA modelling, there are several papers discussing both in the literature.

**Response:** We have conducted a rigorous spatial autocorrelation analysis examine any spatial agglomeration of NCDs spending efficiency across the sub-Saharan Africa. Longitudinal analyses were also carried in the revised manuscript (see pages 10 – 12, 24 – 26, Tables 8 & 10, and Figure 4).

**Comment 3:** Please, provide evidence that results are robust against endogenous clustering and/or other a-priori exogenous clustering.

**Response:** We applied the cluster-robust standard errors, clustering at the country level, in the estimation of the SFA model to correct for any potential heteroscedasticity problem in the data (see pages 20 and Table 6). We want to thank Reviewer #3 for his/her insightful comments. It took us more than two months to learn many new things to be able to satisfy all the comments, but it is worth the effort. We, therefore, acknowledge his/her contribution to this work.

Thank you.

… END …

Thank you.

Yours sincerely,

Kwadwo Arhin & Disraeli Asabte-Darko

Authors
